# Time on task matters most in video game expertise

**DOI:** 10.1371/journal.pone.0206555

**Published:** 2018-10-29

**Authors:** Sebastian Röhlcke, Christian Bäcklund, Daniel Eriksson Sörman, Bert Jonsson

**Affiliations:** Department of Psychology, Umeå University, Umeå, Sweden; Iwate Medical University, JAPAN

## Abstract

In this study, we investigated whether working memory capacity (WMC), personality characteristics (grit) and number of matches played (time on task) can predict performance score (matchmaking rating [MMR]) in experienced players of a popular video game called Dota 2. A questionnaire and four online-based cognitive tasks were used to gather the data, and structural equation modelling (SEM) was used to investigate the interrelationships between constructs. The results showed that time on task was the strongest predictor of MMR, and grit also significantly influenced performance. However, WMC did not play a substantial role in predicting performance while playing Dota 2. These results are discussed in relation to sample characteristics and the role of deliberate practice and skill acquisition within the domain of playing Dota 2. Further, we suggest that future research investigates the social aspects of attaining skill, the relationship between personality and performance, and the qualitative aspects of time spent on a task.

## Introduction

The purpose of this study was to examine working memory capacity (WMC) and personality in relation to performance experienced players in a modern, complex and popular strategy game called Defence of the Ancients 2 (Dota 2). Strategic games have been played all over the world since ancient times, and players who perform well in these games are usually considered smart and astute [1]. Due to the demanding nature of Dota 2, the present study aimed to examine whether high performance in Dota 2 requires higher levels of WMC and a willingness to work hard over time. For decades, scientists have been interested in examining the relationship between cognitive functioning and performance in complex strategy games [2]. Research has shown that skilled performance in classic strategy games like *Chess*, *Mancala* and the *Towers of Hanoi* is associated with intelligence [1, 3–5]. Expert chess players have higher intelligence quotient (IQ) scores than average, and a correlation has been found between chess players’ rank and intelligence [6–7].

The notion that there are associations between cognitive functions and performance in games, such as chess, has been extended to the domain of video games. Rabbit et al. [8] found a modest correlation (*r* = .283) between IQ and initial performance in the game “Space Fortress”. Since then, many papers on this topic have been published covering different subjects regarding video games and cognition (for example, see: [9–13]). In a recent study, the link between gameplay performance in a popular video game called “League of Legends” (LoL) and fluid intelligence was examined. The results showed a correlation between performance on a fluid-intelligence task, the matrix subset from the Wechsler Adult Intelligence Scale–second edition (WAIS-II) and rank in LoL [13]. Another point of interest within this research field is the question of whether playing video games will enhance cognitive abilities, which seems to yield mixed results. Some studies support the idea that playing video games enhances cognitive ability [12, 14–15], while others do not [16–17].

The rapidly growing interest in the relationship between video games and cognition has to do with the identification of video games as complex and cognitively demanding tasks [11]. Thus, video games are being used in psychological research to investigate skill acquisition, cognitive functioning and aging [18].

A specific cognitive construct that is important for learning from experience and adapting to the environment is our *working memory* (WM). There are numerous models that try to define what WM is and how it functions [19]. One of the most influential is *Baddeley’s model of WM*, which describes the cognitive mechanisms that are associated with planning, reasoning, decision making, problem solving, multitasking, focusing attention and learning, among others [20–22]. WM is responsible for the simultaneous maintenance and manipulation of information needed to perform the complex cognitive tasks presented above [20–22]. Baddeley's model of WM contains a central executive and three sub-systems: the phonological loop, the visuospatial sketchpad and the episodic buffer [20, 23]. The central executive is a system that controls attention, and its primary function is to distribute attentional resources to the sub-systems [24]. The phonological loop is a system that briefly stores the signal received through the auditory sensory system. The visuospatial sketchpad is a system that complements the phonological loop, and it is assumed to work via the same principles as the phonological loop, but for visuospatial information [20]. The episodic buffer is the part of the WM that is thought to allow stimuli from different WM subsystems, the central executive and long-term memory (LTM) to interact and unify in a multidimensional code [23]. Research has shown that WM capacity (WMC) shares nearly 60% of its variance with fluid intelligence [25]. Another study showed that WMC is almost perfectly predicted by general intelligence (Standardized beta loading was .96, on average) in a confirmatory factor analysis conducted on the scores obtained from 594 participants taking several cognitive psychometric tests [26].

Recent research on skill acquisition and performance has increased the amount of interest in the combination of cognitive measures and measures of personality [27]. Several different perspectives apply to the understanding of *personality*; thus, there are many definitions and theories encompassing personality, including cognitive, humanistic and sociocultural ones [28]. Over the last ten years, a lot of research has been done to define, develop and examine the predictive value of the personality trait, grit [29]. Grit is defined as the perseverance and passion for long-term goals and includes persistent effort despite setbacks [29]. The presence of long-term stamina, rather than the need for instant rewards, is emphasized by the authors, as is the resistance to a lack of feedback and failure. Hence, grit can predict academic success in Ivy League students, over and above intelligence [29]. Grit has also been shown to be a useful predictor of educational success among military officer candidates [30]. However, research on grit as a predictor of athletic success has shown mixed results [31–32]. Duckworth’s [33] finding show that “gritty” students spend more time studying, which results in better exam performance. Thus, the application of grit on the domain of Dota 2 creates an intriguing avenue through which to aid the investigation of expert performance.

With these considerations in mind, the objective of this study was to examine whether WMC, personality characteristics (grit) and the amount of Dota 2 matches played (time on task) are factors that predict performance score (matchmaking rating [MMR]) among experienced players in Dota 2.

## Method

### Participants

To participate in the study, and to be regarded as experienced Dota 2 player, several criteria had to be met. Participants needed (1) to be 15 years of age or older; (2) to have played at least 110 ranked games of Dota 2; (3) to have played at least ten games during the past month; and (4) to have read and accepted the letter of consent. A total of 353 participants were recruited for the study via Dota-specific Internet communities using an informative homepage that presented the study.

Of these initially recruited participants, 10 participants dropped off during the cognitive tasks; 19 outliers were identified as values outside the 1.5 interquartile range and were thus removed from the dataset. Accordingly, based on the operation span instructions, 20 participants did not meet the criteria where they had to score above 80% on the concurrent mathematical tasks; therefore, these individuals were excluded [34]. Thus, in all, 49 participants were excluded from the statistical analysis, which left a remaining sample size of *n* = 304. Results from Student’s t-test comparing excluded participants with available MMR data (*n* = 44) and the study sample (*n* = 304) revealed no differences with regard to MMR score; *t*(346) = 1.20 *p* = .23.

Participants’ ages ranged between 15 and 38 years, with a mean of 22.5 (*SD* = 4.53) years. In total, 94% percent were men, 4% were women and 2% preferred not to say. The highest degree or level of school completed varied from primary school to a doctorate degree, where 24.2% of the participants had finished high school, 31.3% had taken some college credits, and 26% had a bachelor's degree. A total of 49% of the sample had played games in the same genre before they started playing Dota 2. The participants were from 48 different countries, with every continent represented. From the internet community, population data are available with respect to MMR score [35]. A download of this data and descriptive analyses using 110 games being played (same inclusion criteria as in our sample) as the lower boundary revealed a mean value of 2853.20 with a standard deviation of 913,23, based on 1 515 525 players. The mean MMR score in our sample (*M* = 3323, *SD* = 1302) was higher but within one standard deviation of the mean MMR score in the general Dota 2 population.

The data collection followed the guidelines stipulated in the Declaration of Helsinki. In accordance with the Ethical Review Act of Sweden, no participants were under the age of 15, no sensitive personal data was collected, and data collection did not cause any psychological or physical discomfort to the participants. The cognitive data collected in the study has previously been approved by the Regional Ethics Board at Umeå University (dnr 2015-382-31Ö). The participants were required to read and accept the letter of consent before participating in the study. They were informed that their participation was voluntary and that they could quit whenever they wanted without providing any explanation. Further, they were also informed that they could have their data deleted and that the results would be presented at the group level only.

### Procedure

Data collection took place online with the use of Google Forms and a Web-based, automated cognitive test battery. Participants were first shown a letter of consent together with instructions for the study. Following the letter of consent, the participants filled out their background information, such as age, gender, level of education and country of residence. They also filled out information about their Dota 2 experience, such as MMR and total games played (time on task). Finally, they filled out the short grit questionnaire. At the end of the questionnaire, participants were rerouted to a secure external server where they completed the operation-span, visuospatial-span, and digit-span tasks. A total of 40 minutes was required to complete both the questionnaire and the cognitive tasks.

### Measures

#### Video game description and MMR score

Dota 2 is developed and distributed by Valve Corporation. With over 630,000 average players per hour in 2016, Dota 2 is one of the most popular games of our time [36]. Every year, professional teams compete in a series of tournaments, ending with The International (TI), which is the world championship of Dota 2. In 2017, over 92 million unique viewers tuned in to watch the games at TI, where the top 16 teams competed during a one-week-long event with a prize pool of $24.000.000 [37].

Dota 2 is a free-to-play, match-based strategy game that is played online in real time. The game is played five versus five with the goal of destroying the opponent’s base, and every player controls one of 115 unique characters. The characters have unique sets of abilities and skills that grow stronger during the game, depending on how well players gather and manage the games’ resources: gold and experience. With 152 different items, 115 unique heroes and over 480 distinctive spells, intricate interactions between items, heroes and spells are made available for players to exploit. To succeed in Dota 2, knowledge about these interactions is crucial. Each team has a total of 17 buildings at the start of a game, which is divided into three ‘lanes’ connecting the two bases. The defensive buildings must be destroyed sequentially along these lanes to reach and destroy the enemy base. A typical game of Dota 2 takes about 40 minutes to play and is divided into three different phases: early game (∼ first 10 minutes), mid-game (∼10–30 minutes) and late game (∼30 minutes and longer). These phases introduce specific demands on player decision making, itemization and mechanical skills. Dota 2 is also socially demanding in the sense that players need to communicate, cooperate and coordinate under pressure according to match-unique circumstances.

Each player’s performance in Dota 2 is represented by that individual’s MMR, which is quantified in MMR points. A proprietary algorithm regulates the international matchmaking based on players’ MMR scores [38]. The algorithm works similarly to the ELO system, which is a well-known ranking system that was developed to determine player ranks and the matching of players within the domain of competitive chess [39]. ELO is also used in traditional sports such as soccer [40]. Within ELO systems, higher player ratings indicate higher skill levels. A player’s rating increases and decreases depending on the outcomes of matches versus other rated players. The winner of a match yields rating points from the loser, and the total number of transferred points is determined by the difference between the player’s ratings [39]. In this study, MMR ratings were collected through the online questionnaire that was completed by the participants.

#### Time on task

As part of the online questionnaire, participants filled in the total number of games played in Dota 2

#### Grit

Short Grit Scale (Grit-S) aims to capture perseverance and one’s passion for long-term goals with four fewer items than the original. By answering eight items describing the participants’ tendency to engage in sustained interest (e.g., “New ideas and projects sometimes distract me from previous ones”, reverse scored) and enduring effort (e.g., “I finish whatever I begin”) using a 5-point Likert-like scale (where responses range from 1 = *not at all like me* to 5 = *very much like me*), a measure of an individual's “grittiness” is estimated. There is evidence that Grit-S is a valid and reliable instrument, with acceptable internal consistency (α ranging from .73 to .84 across four samples) and adequate test-retest stability (*r* = .68, *p* < .001) [41].

#### Working memory capacity

In this study, WMC was operationalized using Baddeley’s model of WM and was thus measured with three different tasks. The first was a complex WM task called *operation span* in which participants are presented with a series of interwoven letters and arithmetic tasks. Participants were to alternate between storing series of letters in their short-term memory and performing arithmetic operations. In this study, the dependent variable was the number of letters retrieved correctly. Operation span has good internal consistency (alpha .78) and test–retest reliability (.83) [34]. The second task is called *spatial span*, which is a short-term memory visuospatial task. In this task, participants were first shown a 4 × 4 grid of squares. Next, the squares lit up in a sequence, one by one. The participants were then asked to reiterate the sequence when it ended. To calculate a score, the participant needed to successfully remember which squares were illuminated in the right order for two trials in a row or more. In this study, the dependent variable was the highest number of illuminated squares recalled. The third task is called *digit span*, which is a short-term phonological task. In this task, participants were presented with a sequence of single digit numbers (ranging from 1 to 9). When the sequence ended, participants had to use their keyboard to repeat all the numbers in the same order as they appeared on the screen. In this study, the dependent variable was the highest amount of numbers recalled correctly.

#### Covariates

Age and level of education were used as covariates in the analyses.

### Statistical analyses

First, a correlation analysis was performed on the variables included in this study and Pearson’s coefficient was used. Next, structural equation modelling (SEM) was used to assess the relationship between WMC, grit (Grit-S) and number of matches played (time on task) in Dota 2 performance (MMR). Three fit indices were used to evaluate each model: Bentler's comparative fit index (CFI), the root-mean-square error of approximation (RMSEA), and chi-square divided by the degrees of freedom. To explore how each variable was associated with MMR, standardized parameter estimates were used. Data were analysed with SPSS-25 and AMOS-23 using the maximum likelihood method.

## Results

Overall, the findings showed that the number of matches played (time on task) was the strongest predictor of performance (MMR). Further, the results showed significant effects of grit and age as predictors, but no effects of working memory capacity (WMC). Descriptive data (Table 1), followed by SEM modelling of the independent and dependent variables are presented below.

**Table 1:** Characteristics of the sample used in the analysis.

	Mean	SD	Skewness^a^	Kurtosis^b^
1. Age	22.5	4.5	0.57	-0.08
2. Education	3.9	2.0	0.59	-0.70
3. Time on task	2759.7	1726.4	0.74	0.03
4. Operation span	49.4	14.7	-0.33	-0.46
5. Spatial span	15.1	3.3	-0.05	-0.22
6. Digit span	3.9	1.5	-0.11	-0.03
7. Grit	3.0	0.7	-0.06	-0.55
8. MMR	3323.2	1302.5	0.06	-0.32

*Note*. MMR = Matchmaking rating.

^a^Std. Error = .14.

^b^Std Error .28.

**Fig 1:**
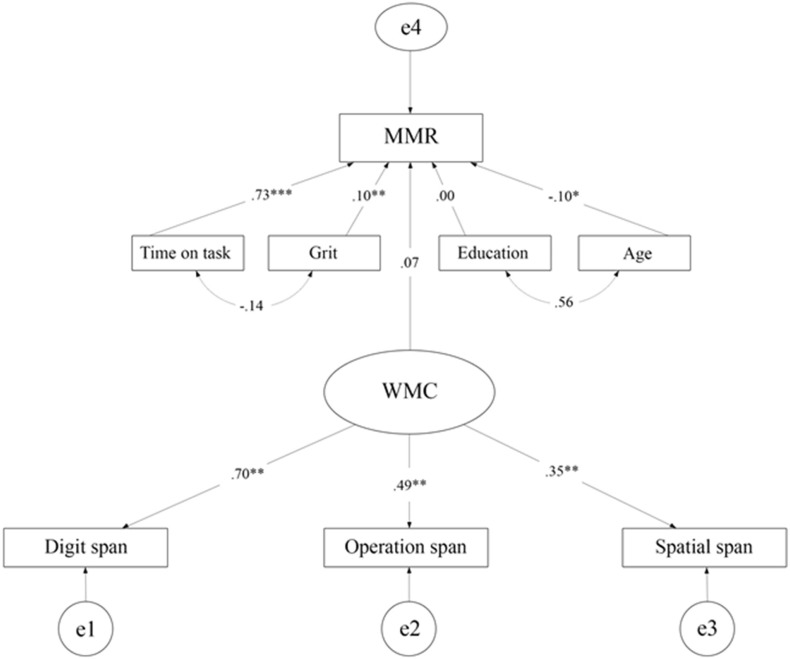
Structural equation model of the effects of working memory capacity (WMC), grit (Grit-S), time on task (number of matches played), age and education on performance in Dota 2 (MMR). *** indicates *p* < .001, ** *p* < .01, * *p* < .05. Significant correlations between predictor variables are illustrated by double headed arrows.

As can be seen, the values of skewness and kurtosis indicated normally distributed data for all variables included in the analysis. Next, we examined fit indices of the structural equation model that included WMC (operation span, spatial span and digit span), the personality trait grit (Grit-S), the number of matches played (time on task), age and level of education as predictors of performance in Dota 2 (MMR); see Fig 1.

The model indicated good fit (*CFI* = .96, *RMSEA* = .06, χ^*2*^/*df* = 1.93). For the CFI, values greater than or equal to 0.95 indicates acceptable model fit [42]. For RMSEA, a value of .06 or less is equal to good model fit, whereas a value of .08 indicates a reasonable fit [42–43]. For normed chi-square values, the upper thresholds differ between 2.0 [44] and 5.0 [45] in the literature. Thus, the CFI, RMSEA and the normed Chi-square values were indicative of a good model fit. Standardized regression weights together with *p*-values and Bayes factors (BF) from predictors included in the analyses are provided in Table 2.

**Table 2:** Regression weights and Bayes factors of predictors in the structural equation model with matchmaking rating as dependent variable. Column six (BF *H*_1_ vs *H*_0_) display the probability that the alternative hypotheses (*H*_1_) are correct while column seven (BF H_0_ vs H_1_) display the probability that the null hypotheses (*H*_0_) are correct.

	β	B	S.E.	*P*	BF H_1_ vs H_0_	BF H_0_ vs H_1_
WMC → MMR	.07	82.74	66.75	.215	0.48	2.08
Age → MMR	-.10	-30.49	13.69	.026	3.86	0.26
Education → MMR	0	1.89	30.44	.951	0.37	2.72
Grit → MMR	.10	209.42	78.71	.008	9.21	0.11
Time on task → MMR	.73	.56	.03	.000	75.45^45^	< 0.00

*Note*: β = Standardized regression weight, B = Unstandardized regression weight, S.E. = Standardized error of B, BF = Bayes factor, MMR = Matchmaking rating, WMC = Working memory capacity

As can be seen, time on task has the greatest explanatory value of the predictors, whereas grit and age had a small (as indicated by the standardized regression weights), but significant, influence on the MMR score. WMC and education were not significant predictors of performance in Dota 2. Results of the BF analyses suggest that *H*_1_ are more likely than *H*_0_ for Time on task, grit, and age_._ Thus, results from Bayesian analyses give further strength to the findings.

### Additional data collection and analyses

**Fig 2:**
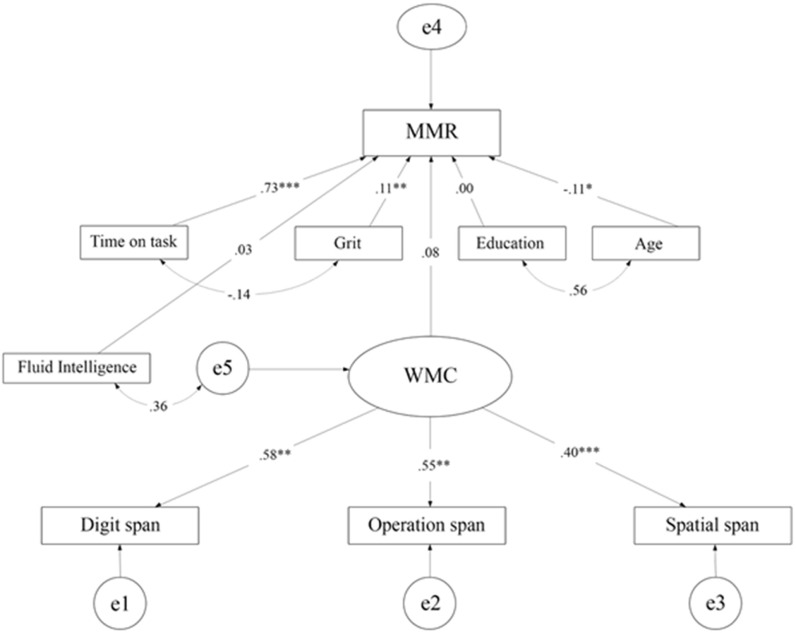
Structural equation model of the effects of working memory capacity (WMC), grit (Grit-S) time on task (number of matches played), fluid intelligence (Raven's matrices), age and education on performance in Dota 2 (MMR). *** indicates *p* < .001, ** indicates *p* < .01, * indicates *p* < .05. Significant correlations between predictor variables are illustrated by double-headed arrows.

The non-significant effect of WMC raised the question of whether other cognitive measures would yield a different result (c.f. [13]). It was thus decided to include a measure of general fluid intelligence (*Gf*) as a predictor of MMR. During additional data collection, all participants included in the main analyses were asked to take the Raven advanced progressive matrices test [46] as a measure of *Gf*. Of the 304 participants who completed the study, 118 participants signed up and took the test. One outlier was identified and removed from the dataset, which resulted in a subsample of *n* = 117, where the values of skewness (-.50) and kurtosis (-.35) indicated normally distributed data. To investigate whether the subsample differed significantly from the total sample with respect to all dependent variables we conducted independent *t*-tests, (all *p*-values > .25). This model complemented the model used in the main analysis with a measure of *Gf* as a predictor variable. However, the results showed that fluid intelligence (*β* = -.03, *B* = 12.68, *SE* = 25.61, *p* = .62, BF_10_ = 0.42, BF_01_ = 2.34) did not add any explanatory value to the model of predicting MMR, whereas grit, age and time on task were still significant predictors of MMR (see Fig 2).

## Discussion

The goal of the present study was to examine WMC, grit and time on task as predictors of player performance among experienced Dota 2 players. In total, 304 participants were included in the statistical analysis. Within this sample, 117 later completed a version of Raven's matrices in the second round of data collection. The SEM analyses showed that time on task was a strong predictor of performance in Dota 2. Neither WMC nor fluid intelligence was significant. In contrast, grit was found to be a significant predictor. The present study indicated that time on task and, to some extent, the personality characteristic grit were important for Dota 2 skills.

The results from this study revealed no relationship between WMC and MMR; however, these results are in line with earlier research, which also failed to find a significant correlation between WMC and performance within the multiplayer online battle arena (MOBA) genre [13]. Further, the results showed that fluid intelligence had no significant relationship with MMR, which was unexpected since earlier research has shown a moderate and significant correlation between fluid intelligence in a commercial video game in the same genre [13]. However, it is still possible that WMC and fluid intelligence may have some influence on performance in Dota 2. Learning curves often are disproportionately important during the learning process [47], and it is possible that our sample overall consisted of participants with relatively good WMC and fluid intelligence that had a fast learning curve at the beginning of playing the game, whereas individuals with low WMC and fluid intelligence instead quit playing early due to slow learning progress, and therefore did not fulfil the criteria’s to participate in the present study. The sample examined has as pointed out a mean MMR score that was higher than the population. However, notably within one standard deviation, meaning that the obtained sample was marginally better with respect to MMR scores and can, therefore, be regarded as a relatively representative sample of the general Dota 2 population.

The results showed a small but significant relationship between grit and MMR. This favours the conceptual idea that high performance in Dota 2 requires passion and perseverance for long-term goals. In addition to predicting academic, sports and job performance [31–32, 48], our results indicate that grit might be important for understanding performance within the domain of video games as well. Our results are further in line with findings that show how grit only moderately correlates with performance [49]. It is, however, possible that the effects of grit would have been stronger if we had included beginners of Dota 2 into the analyses. That is, experienced individuals may have smaller differences with regard to the grit factor. Duckworth [41] showed that there was a relationship between grittiness and time dedicated to studies within academia which, in turn, predicts academic performance. However, our study shows a negative relationship between grittiness and time on task. Within Dota 2, operant conditioning in terms of intermittent rewarding components might override a lack of grittiness. It is possible that Dota 2 players do not perceive setbacks as real failures since the system is adaptable and introduces new reinforcements during a game.

Notable is that grit in a recent meta-analysis [49] was found to be highly correlated with the Big 5 subscale conscientiousness and the predictive power of grit over and above conscientiousness was therefore questioned. In comparison to conscientiousness, there is a restriction of range with respect to the number of items in the grit survey. A correlation between conscientiousness and MMR could potentially therefore been slightly changed compared to the correlation between conscientiousness and grit found in the present study.

It is possible that also other personality factors may have influenced the results. For instance, the relationship between time on task and the MMR score may to some extent be confounded by motivational factors and self-belief. Studies have found expectancy and value beliefs of an activity to be important components of engagement in sports (see e.g., [50–52]). According to the expectancy-value model of achievement choice, the belief of how well we will do (expectancy-related beliefs) and how much we value an activity (task values) are important predictors of our choice, persistence, performance, and effort to reach success. Thus, the perceptions of our current competence and our expectancies of success in the future are important factors to consider when predicting performance in sports. It is, therefore, possible that individuals with higher expectancies and belief of success in Dota 2 (which to some extent also may be accurate) are the ones more likely to spend more time on a task which in turn is related higher MMR scores.

The present study showed that time on task was the strongest predictor of MMR among experienced Dota 2 players. This result is in line with the acquired skill explanation of expert performance (e.g., [53]). Ericsson, Krampe, and Tesch-Römer [54] introduced the concept of *deliberate practice* and argued that it is critical to take the qualitative aspects of practice into consideration when investigating skill acquisition. In short, deliberate practice can be defined as systematic and purposeful training that requires focused attention and has the specific goal of improving performance. By definition, it is thus distinguished from both working and playing. As part of their argument, Ericsson et al. [54] demonstrated that the skill improvements of an individual who only spends time doing one task would eventually reach a plateau. Thus, the authors argue that deliberate practice is necessary to overcome a stagnated learning curve. The findings in the present study showed that time on task is the critical component when predicting MMR. It is also possible that participants scoring high on grit are more likely to engage in deliberate practice. Therefore more gritty participants become more successful in a shorter time [55], hence the negative correlation between grittiness and time on task found in the present study.

However, from our data, we cannot conclude that deliberate practice among players is underlying the relationship between time on task, grit, and MMR. It is, of course, possible that deliberate practice occurs but, as pointed out above, it is also possible that the operant conditioning feedback embedded in the game is enough to promote increased skill, thus requiring very little deliberate practice. This reasoning is in line with a meta-analysis that concluded how deliberate practice is relevant, but its effectiveness is not as strong as Ericsson and his colleagues claim. Macnamara, Hambrick, and Oswald [56] argued that future research needs to consider innate individual differences as important predictors when investigating expert performance, since deliberate practice only accounts for 26% of the variance in skill within different games. Our finding indicated that time on task was the strongest predictor of performance in Dota 2, while measures of cognition were non-significant. It should be stressed though, that a significant component of cognition–including intelligence–involves rapid learning, successful action and decision making in novel situations, which is when the time is scarce, and the information is low. Computer games might be different because they are so well-structured and have clear and definitive (albeit not explicit) rules, such that performance in Dota 2 might rely very much on experience, which is in-line with the findings from the present study.

In this study, and as opposed to a laboratory setting, the participants took part in the experiment from a computer at home. Thus, it was not possible to ensure that the instructions were followed. On the other hand, Web-based data collection allowed us to reach out to a young, global and online-based community to collect a large sample in a reliable way. This assumption is in line with previous studies showing that Web-based studies can be reliable [57]. The use of self-report measures does, of course, include risks such as social desirability bias and the potential to misunderstand the questions asked. However, this is not unique for the present study, as these challenges are always present when distributing surveys and self-report measures.

This study presents novel evidence but needs to be replicated in future studies. Further research should use the large amounts of data that are made available on digital platforms since these platforms can make it possible to assess research on a number of intriguing questions related to skill acquisition [18].

This study concludes that the numbers of matches played matters the most when explaining player performance within the domain of strategic, team-based video games such as Dota 2. Conversely, cognition does not play a vital role for player performance, and the personality trait grit only plays a small role in its explanation.

## Supporting information

S1 File Data used in the present study. (XLSX)
